# Quantification of Calcified Particles in Human Valve Tissue Reveals Asymmetry of Calcific Aortic Valve Disease Development

**DOI:** 10.3389/fcvm.2016.00044

**Published:** 2016-11-04

**Authors:** Katsumi Yabusaki, Joshua D. Hutcheson, Payal Vyas, Sergio Bertazzo, Simon C. Body, Masanori Aikawa, Elena Aikawa

**Affiliations:** ^1^Division of Cardiovascular Medicine, Center for Interdisciplinary Cardiovascular Sciences (CICS), Brigham and Women’s Hospital, Harvard Medical School, Boston, MA, USA; ^2^Department of Medical Physics and Biomedical Engineering, University College London, London, UK; ^3^Center for Perioperative Genomics, Brigham and Women’s Hospital, Boston, MA, USA; ^4^Department of Anesthesiology, Brigham and Women’s Hospital, Boston, MA, USA

**Keywords:** particle labeling, image analysis, calcification, particles, microcalcification, atherosclerosis, calcific aortic valve disease

## Abstract

Recent studies indicated that small calcified particles observable by scanning electron microscopy (SEM) may initiate calcification in cardiovascular tissues. We hypothesized that if the calcified particles precede gross calcification observed in calcific aortic valve disease (CAVD), they would exhibit a regional asymmetric distribution associated with CAVD development, which always initiates at the base of aortic valve leaflets adjacent to the aortic outflow in a region known as the fibrosa. Testing this hypothesis required counting the calcified particles in histological sections of aortic valve leaflets. SEM images, however, do not provide high contrast between components within images, making the identification and quantification of particles buried within tissue extracellular matrix difficult. We designed a new unique pattern-matching based technique to allow for flexibility in recognizing particles by creating a gap zone in the detection criteria that decreased the influence of non-particle image clutter in determining whether a particle was identified. We developed this flexible pattern particle-labeling (FpPL) technique using synthetic test images and human carotid artery tissue sections. A conventional image particle counting method (preinstalled in ImageJ) did not properly recognize small calcified particles located in noisy images that include complex extracellular matrix structures and other commonly used pattern-matching methods failed to detect the wide variation in size, shape, and brightness exhibited by the particles. Comparative experiments with the ImageJ particle counting method demonstrated that our method detected significantly more (*p* < 2 × 10^−7^) particles than the conventional method with significantly fewer (*p* < 0.0003) false positives and false negatives (*p* < 0.0003). We then applied the FpPL technique to CAVD leaflets and showed a significant increase in detected particles in the fibrosa at the base of the leaflets (*p* < 0.0001), supporting our hypothesis. The outcomes of this study are twofold: (1) development of a new image analysis technique that can be adapted to a wide range of applications and (2) acquisition of new insight on potential early mediators of calcification in CAVD.

## Introduction

Cardiovascular calcification is a leading predictor of and contributor to morbidity and mortality (1, 2). Calcification progresses as an active disease that is regulated by the balance between factors promoting and inhibiting mineralization. Two distinct forms of cardiovascular calcification predominate clinically – atherosclerotic vascular calcification and calcific aortic valve disease (CAVD) (3, 4). Size and morphology determines the impact of calcification on these cardiovascular tissues (5–10). We recently showed that in pathologic conditions cells within atherosclerotic plaques release specialized extracellular vesicles (~100 nm) that nucleate calcium phosphate mineral (11, 12). During the calcification process, these vesicles aggregate and fuse to form microcalcifications, which can then proceed to form larger calcifications. While larger calcifications (>30 μm) may stabilize atherosclerotic plaques (13–16), small microcalcifications (~5 μm) that form within vulnerable atherosclerotic fibrous cap contribute to plaque rupture by inducing high biomechanical stress adjacent to the hard mineral in the otherwise soft tissue (7, 17). Myocardial infarction or stroke occurs due to the resultant occlusion of the artery lumen. In aortic valve leaflets, microcalcifications may not significantly affect valvular function; however, the formation of large calcifications impairs biomechanical integrity (18). One distinguishing feature of CAVD is the asymmetric initiation and progression of calcification during disease development. Calcification begins at the base of aortic valve leaflets, near the attachment to the aortic wall. Furthermore, the calcification localizes on the aortic aspect of the leaflets in the collagen-rich fibrosa. The reason for the asymmetric presentation of CAVD remains unknown.

Scanning electron microscopy (SEM) provides the resolution to visualize all calcification morphologies and sizes and has been utilized to identify different types of calcification in cardiovascular tissues. Recently, SEM analyses have identified an abundance of previously unidentified calcified particles ranging in diameter from 100 nm to 5 μm in cardiovascular tissues (19). These particles were observed not only in diseased regions but also in regions with no obvious gross pathology. This suggests that these particles may presage gross calcific remodeling and should be further studied to understand their potential role in disease initiation (19). We hypothesized that if these particles precede CAVD, the regional distribution of the particles should exhibit the same asymmetry observed in leaflet calcification. Although methods to characterize the chemical makeup of these particles have been developed, reliable recognition and quantification of these nano-size particles from SEM tissue images is difficult. Calcified particles in images obtained from different regions of tissues exhibit a heterogeneous range of numbers, size, shape, and brightness within a complex and noisy extracellular matrix tissue background. Therefore, methods to quantify calcified particles within these images must exhibit flexibility to overcome the inherent noise.

ImageJ is commonly used for particle detection (20–22). The preinstalled basic particle-labeling (BPL) method consists of three steps: (1) convert the grayscale or color image to a black and white binary image using a predetermined threshold value, (2) extract all particulate objects in the image, and (3) label particles that fulfill target parameters (size/shape). Template matching/pattern matching is another conventional method that seeks target objects in test images through comparison with a template image (23, 24). Objects in the test image are labeled as targets, if they exhibit similarity to the template as determined by an assigned threshold value.

We established a novel particle-labeling method based on the pattern-matching technique. This approach uses a unique pattern, which allows flexibility in recognizing particles with variable size, shape, and brightness within a noisy tissue background of a single SEM image. This method resulted in a more robust and accurate particle detection than conventional BPL and template-matching approaches and outperformed the time-consuming and error-prone manual quantification. In this paper, we developed and demonstrated the efficacy of this new method in detection and quantification of calcified particles in synthetic test images and SEM images of human atherosclerotic plaques. We then applied our new method to quantify the geometric distribution of calcified particles in histological sections of human aortic valve leaflets. Our results indicate that the calcified particles predominately localize at the base of the valve leaflets within the fibrosa layer, matching the asymmetry observed in gross CAVD remodeling. These particles may serve as precursors to the formation of microcalcifications and subsequent larger calcifications, either by direct contribution to mineral formation or by stimulating osteogenic differentiation of local cells. Future work is needed to further clarify their relevance to CAVD.

## Materials and Methods

### Scanning Electron Microscopy Imaging of Human Cardiovascular Plaque Samples

Human carotid atherosclerotic samples were obtained from patients undergoing endarterectomy. Human aortic valve leaflets were obtained from aortic valve replacement surgeries. All tissue samples, including aortic valves and atherosclerotic plaques that had common morphological features of calcified unstable atherosclerotic lesions, were de-identified, and no inclusion or exclusion criteria were used for this study. All tissue collection protocols were approved by the Institutional Review Board at Brigham and Women’s Hospital (2011P001703). The tissue samples were washed in PBS upon collection and embedded in optimal cutting temperature compound. Frozen tissue blocks were stored at −80°C before sectioning using cryomicrotome. Tissue sections of 15 μm thickness were obtained and fixed in a 4% (w/v) paraformaldehyde (Sigma) solution in phosphate buffered saline (PBS; Sigma).

For SEM analyses, samples were secured to an aluminum sample holder with carbon tape, and silver paint was applied to the area immediately surrounding each sample, which was then coated with 5 nm chromium in a sputter coater (Quorum Technologies Sputter Coater model Q150T S). Following the coating procedure, samples were imaged by SEM (Gemini 1525 FEGSEM), operated at 10 kV using the backscattering and secondary electron image mode acquisition. The SEM images were taken with 2,000× magnification to allow visualization of small calcified particles. Seven images from random regions of the tissue sections were obtained from each of two independent donor carotid endarterectomy samples and three aortic valve donor samples.

### Preparation of Synthesized Particle Image

Computationally synthesized particle images were used to design unique particle-matching patterns that allow flexibility to recognize heterogeneous particles. The images included gray to white dots with varied sizes (2–16 pixels in height) and shapes (circle to oval) in artificially created noisy backgrounds (black background, gradually changing gray scale background or squared blocks with different brightness). Appropriate synthetic background levels were determined by referring to the actual background levels in the SEM images.

### Image Particle Counting Method Using ImageJ and Custom Software

First, we counted calcified particles in SEM images using the ImageJ software (version 1.45S, National Institute of Health, USA) equipped with a particle-labeling function. We took the procedure below as the BPL method, using several processes preinstalled in ImageJ, namely, convert the color mode of the image to grayscale (8-bit depth), apply a predetermined threshold value (0–100) of brightness to convert each grayscale image to a black and white binary image, label particles (3–200 pixels in area) and unchecked “Exclude on edges” and “Include holes” options. Some parameters (threshold value for binarization and lower limit of particle size) were critical to detect particles accurately. The optimal parameter values were determined prior to the analysis (Figure S1 in Supplementary Material).

To extend the BPL analyses, we also evaluated the efficiencies of image preprocessors. We applied “Subtract Background” or “Find Edges” to the image prior to performing BPL to reduce background artifacts/textures derived from the tissues and extracellular matrices and enhance contours of particles, respectively. For “Subtract Background,” we set parameters as follows: rolling ball radius = 20 pixels; checked on disable smoothing. We also developed a custom software tool to analyze particle numbers using the Microsoft Visual Studio 2013 C# software developing environment and AForge.Net Framework. The application software works on Windows-based computers and is available from our website (http://cics.bwh.harvard.edu/).

### Evaluating Particle Recognition Efficiencies of the Image Particle-Labeling Methods Compared with Manual Particle Counting

Target particles (white dots in synthesized test images or calcified particles in SEM images) were manually marked with red dots to make reference dot images. The same images were then examined by the particle-labeling methods. A cyan dot was placed on the image, when the particle-labeling method marked a particle at the same position with a manually identified reference dot. The particles that did not match the reference dots were marked light green. To evaluate particle detection performance of each particle-labeling method, the numbers of matched particles (cyan), false negative particles (red), and false positive particles (light green) were counted to calculate ratio values given by the Eqs 1–3. In the equations *N*_color_ indicates the number of particles labeled with any of the three colors.

(1) $$\begin{array}{l} \text{Detection rate }\left(\%\right)=\frac{N_{\text{cyan}}}{N_{\text{cyan}}+N_{\text{red}}}\times 100 \end{array}$$

(2) $$\begin{array}{l} \text{False negative rate }\left(\%\right)=\frac{N_{\text{red}}}{N_{\text{cyan}}+N_{\text{red}}}\times 100 \end{array}$$

(3) $$\begin{array}{l} \text{False positive rate }\left(\%\right)=\frac{N_{\text{green}}}{N_{\text{cyan}}+N_{\text{green}}}\times 100 \end{array}$$

## Results

### Significant Increase in Particle Recognition by the Combinational Use of Flexible Pattern and Picture Series with Different Sizes

**Figure 1:**
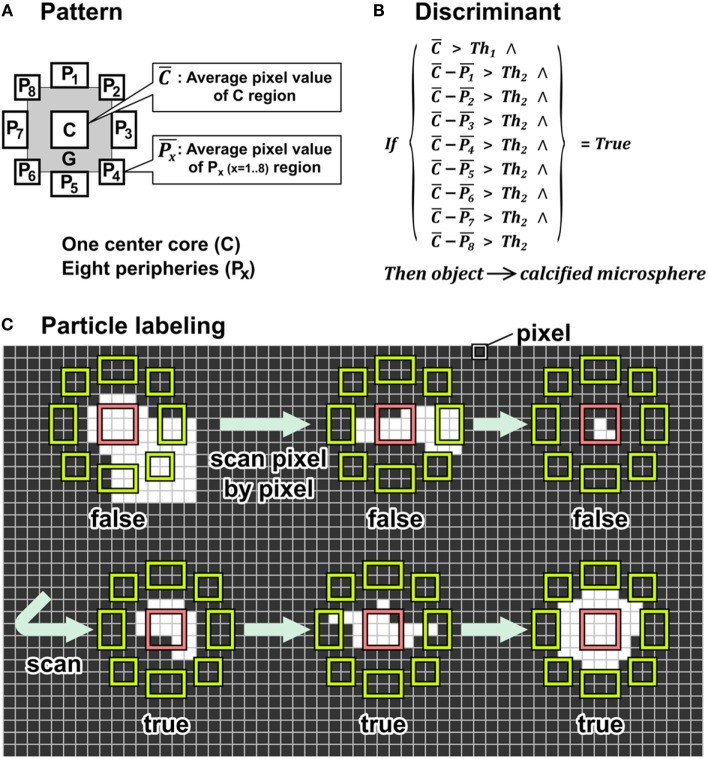
**Algorithm of the flexible pattern particle-labeling (FpPL) method**. **(A)** FpPL used unique pattern consisted of three parts: center core (C) eight peripheries (*P*_1_–*P*_8_), and buffer zone/gap (G). **(B)** Formula of discriminant to determine target object. **(C)** Pattern scanned a test image pixel by pixel. The object was labeled as a target particle when discriminant criteria were met.

We established a novel particle-labeling method based on a pattern-matching technique. In SEM images, calcified particles appear as distinct white dots having brighter pixels than peripheral pixels due to the mineral in the particles. Utilizing this discriminating image property of calcified particles, we developed a unique pattern that differentiates the particles from other features in the periphery. As shown in Figure 1A, we designed patterns with a center core consisting of a 3 × 3 pixel array shown as “C” and eight peripheral areas consisting of 2 × 3, 3 × 2, or 2 × 2 pixel arrays shown as “*P*_1_” to “*P*_8_.” A gap zone labeled “G” separated the core and peripheral areas. The gap zone expands flexibility in particle recognition as pixels in this zone do not affect the decision of the particle detection. This flexible pattern particle-labeling (FpPL) method determines whether any object within the 11 × 11 pixel array can be classified as a particle using criteria shown in Figure 1B and Discriminant 1. The discriminant consisted of two conditional parts: (1) evaluate if the brightness of the center core $\text{(}\bar{C}\text{)}$ is higher than a threshold value 1 (Th_1_) and (2) evaluate if the brightness differences between the center core $\text{(}\bar{C}\text{)}$ and each of the eight peripheries $\left({\bar{P}}_{\text{1}}-{\bar{P}}_{\text{8}}\right)$ is higher than a threshold value 2 (Th_2_). Examples illustrating FpPL particle detection demonstrate implementation of these criteria (Figure 1C). In Figure 1C, all pixel values are shown as black or white for the convenience; however, FpPL uses grayscale pixel values (range: 0–255) to determine the discriminant in actual SEM images. The pattern moved pixel by pixel to scan the whole image from top-left pixel to bottom-right pixel.

Discriminant 1 utilized the difference between pixel intensities in the center and the peripheral zone of the target to quantify calcified particles within SEM images of human carotid arteries. Detailed information about these methods is described in Supplementary Material.

Discriminant 1. Uses differential value between center core and peripheries:

IfC¯>Th1^C¯−P¯1>Th2^C¯−P¯2>Th2^C¯−P¯3>Th2^C¯−P¯4>Th2^C¯−P¯5>Th2^C¯−P¯6>Th2^C¯−P¯7>Th2^C¯−P¯8>Th2^=trueThen object→calcified microsphereElse object≠calcified microsphere

**Figure 2:**
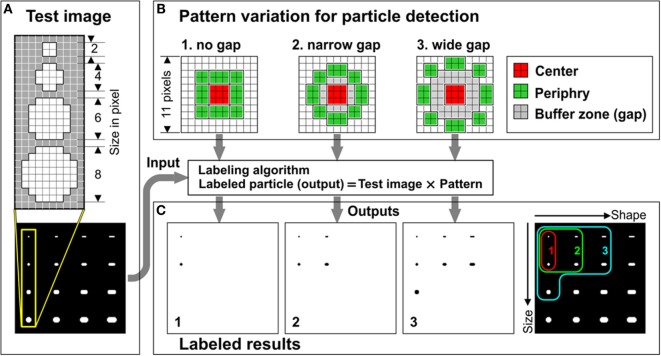
**Buffer zone played an important role in increasing flexibility in particle detection**. **(A)** Dot structures designed in test image. **(B)** Structures of three patterns used in FpPL that had different gap sizes. **(C)** Labeled results (left three panels) and merged result from the three (right panel). Colored curves indicate detectable range of particles by each pattern.

We hypothesized that the gap would allow for flexibility in particle recognition. We first tested this hypothesis using test images of 16 white dots with varying shape and size on a black background (Figure 2A). We prepared three different patterns that had different gap sizes (Figure 2B, “no gap,” “narrow gap,” or “wide gap”). The pattern with no gap showed narrower particle recognition and marked only two dots (Figure 2 × 2 and 4 × 4 pixel sizes). The ranges of detectable particle sizes and shapes were expanded by increasing gap size (Figure 2C). The gap zone allows identification of particles larger than the 4 × 4 core but within the periphery zone. The narrow gap pattern correctly identified 4 out of 16 particles and the wide gap pattern correctly identified 7 out of 16 particles. Even though we also examined gaps wider than two pixels, we chose two pixels for the gap since the patterns with wider gaps tended to show lower detection rate in small particles and higher error rate by detecting non-spherical particles.

Actual calcified particles in SEM images include larger and wider particles than the “wide-gap” pattern could detect in Figure 2C. Therefore, even though our novel pattern had flexibility in particle recognition, a single such flexible pattern would be inefficient in recognizing calcified microspheres when applied to SEM images.

Using multiple patterns on the target image could expand the particle recognition range; however, this approach would need to be optimized for each image to encompass all possible sizes and shapes and would require enormous time to apply and analyze all of the matching patterns to the image. Instead, we used multiple scalings of the images rather than applying multiple patterns. Multi-scale images were generated from the original SEM image by expanding or shrinking the image and a single wide-gap pattern was applied to these images. We hypothesized that this method would deliver similar particle detection results as a multiple-pattern method. Larger particles that the pattern missed in the original image could be labeled in the smaller scale images and particles smaller than the detection resolution in the original image could be labeled when magnified in the enlarged image.

**Figure 3:**
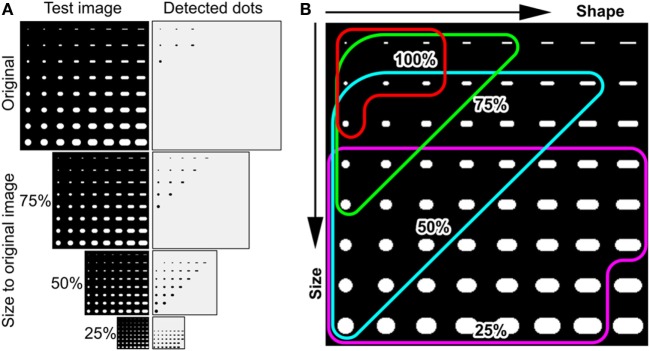
**Using multiple scaled images increased particle detection rates**. **(A)** Test images with multiple scaling shrunk from original image (left) and labeled results (right). **(B)** Colored curves show detectable range of particles in each image size.

To test this hypothesis we applied the wide gap pattern on four different size test images with scales ranging from 100% (original image) to 25% with 25% step (Figure 3A). The pattern recognized larger and wider particles in the smaller scale images. In the smallest image (25%), false positive detection of non-target objects was observed. Therefore, for the identification of calcified particles, we set the scale range from 200% down to 50% to avoid counting unintended larger particles (Figure 3B).

### Flexible Pattern Particle Labeling Provides More Robust and Accurate Particle Detection than Other Conventional Methods

We next evaluated the labeling accuracy of FpPL compared with the other standard methods in ImageJ by analyzing two synthesized dot images with complex background noise (Figure S2 in Supplementary Material). The first test image contained 144 dots in an array (12 × 12) consisting of 3 different sizes and brightness ranging from gray level (pixel value = 145) to white level (pixel value = 255). These dot arrays were mapped on 4 gray scale gradient bands to provide backgrounds of white to black, light gray (pixel value = 43) to dark gray (pixel value = 232), dark to light gray, or black to white (image 1, Figure S2A in Supplementary Material). The other image (image 2, Figure S2B in Supplementary Material) used the same dot pattern placed in image 1, but each dot was mapped on a square of random brightness ranging from almost black (pixel value = 2) to almost white (pixel value = 247). We compared the FpPL particle detection to orthodox particle-labeling methods in these complex images. The first conventional method employed the BPL method preinstalled in ImageJ. We also utilized two image preprocessors prior to performing BPL. “Subtract Background” preprocessor (SBP) was also pre-equipped in ImageJ and could reduce false positive detection by erasing background artifacts/textures in the image. The “Find Edges” preprocessor (FEP) (also preinstalled in ImageJ) can increase detection rate by enhancing the contours of small fuzzy particles. Before beginning the analyses of particles, an initial filtering removed particles with a lower pixel value than the peripheral area, as our target microspheres in SEM images are brighter than the background. The detection rates (cyan dots = matched dots) of SBP and FEP were higher than those of BPL, and error detection rates (red dots, false negatives; light green dots, false positives) were lower than those of BPL in both test images. These results indicated that the preprocessors successfully removed noise and/or enhanced particle contrast. FpPL showed the highest accuracy of the four methods (100% detection rate for image 1 and 99% for image 2).

### FpPL Accurately Detected Calcified Particles Observed in Human Carotid Artery

**Figure 4:**
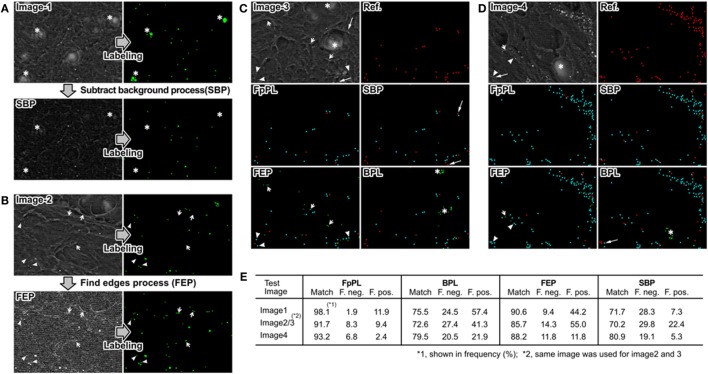
**Error detection in calcified particle identification and effect of image preprocessing**. **(A)** SEM backscattering image 1 shows the presence of large holes that are detected by the BPL method. SBP entirely erased hole-like structures (asterisks) resulting in a large decrease in false positives. **(B)** SEM backscattering image 2 shows that small particles (arrow heads) are often missed by BPL. FEP enhances contrast to allow detection of these small particles but also leads to false positives from the edges of other structures in the image (short arrows). **(C,D)** FpPL reduced false positives (green dots) and false negatives (red dots) and increased accurate particle detection (cyan dots) in images with hole-like structures and sharp edges. **(E)** Detection rate (%) of each method is shown for images 1–4.

Images 1–4 in Figure 4 show typical SEM images of a human carotid artery histological section. The goal of these analyses was to test and optimize the FpPL particle detection algorithm on complex tissue sections prior to aortic valve assessment. Therefore, random areas adjacent to large calcifications were used for analyses. Two artifact structures were observed in many cross sections – large hole-like structures and complex mesh like structures. Each large hole-like structure (asterisk, Figures 4A,C,D) included a nebulous white region in its center and was falsely recognized as a calcified microsphere by BPL. SBP removed this hole, preventing false positive detection (Figure 4A, bottom). FEP enhanced particle contour, resulting in improved detection of smaller and darker calcified microspheres (arrowhead, Figure 4B); however, FEP also increased false positive detection due to enhanced edges of the complex background mesh (short arrows, Figures 4B,D). SBP decreased the brightness level of calcified microspheres, reducing the detection rate (long arrow, Figures 4C,D). The particle-labeling results of the four methods were compared with reference particles labeled by eye (Figures 4C–E). FpPL showed more accurate detection with more matched particles (blue) and fewer errors (false negative particles colored red and false positive particles colored green) than the other three methods in both images (images 3 and 4).

**Figure 5:**
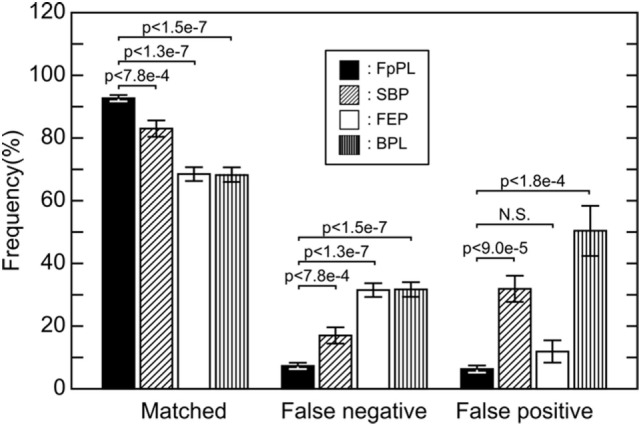
**Detection rate and error detection of particle-labeling methods**. Matches, false negatives, and false positives from 14 SEM images taken from carotid artery sections using 4 methods were examined. Black bars, FpPL; hatched bars, “Subtract Background” process treated prior to labeling; white bars, “Find Edges” process treated prior to labeling; and striped bars, BPL. Values are presented as mean ± SE.

We next examined the labeling accuracies of the methods. We prepared three images with different scalings (200, 100, and 50%) for FpPL to detect calcified particles. As shown in Figure S3 in Supplementary Material, the enlarged image (200% scale) allowed the detection of very small calcified particles (smaller than 300 nm). These very small particles were recognized as calcified particles by eye but FpPL could not detect them in the original image (arrowheads, Figure S3A in Supplementary Material). Using the enlarged image (200%), FpPL accurately detected very small and/or low-contrast dots not recognized by BPL (arrowheads, Figure S3B in Supplementary Material), indicating that FpPL can detect very small particulate objects in noisy backgrounds more accurately than BPL. The results analyzed from 14 SEM images of human carotid artery sections (including 2 different donors) are demonstrated in Figure 5. FEP increased the detection rate (83.0%) compared with BPL (68.3%). FpPL, however, showed a significantly higher detection rate (92.7%, *p* < 0.0000002) and fewer false negatives (7.3%, *p* < 0.0000002) and false positives (6.3%, *p* < 0.0002) than BPL. Even though SBP effectively decreased false positive detection, false negative detection was not improved. This inconsistency is due to the tendency of SBP to reduce the brightness level of calcified microspheres, especially in small particles. FEP reduced both false negative and false positive detection, but it generated new false positives by the over-enhancement of the edges of complex background mesh structures. We next evaluated size-dependent particle detection rate, as we hypothesized that the major increase in detection rate would be caused by an improvement to label very small calcified particles. The results also showed that FpPL had higher sensitivity in smaller particle detection than BPL (Figure S4 in Supplementary Material).

### FpPL Showed More Rapid and Precise Particle Detection than the Multiple Template-Matching Method

Our novel pattern-matching method, FpPL, displayed increased accuracy in particle detection due to its higher sensitivity to detect very small particles and higher robustness against background noise compared with orthodox-labeling methods. In general, pattern-matching techniques can identify unique shapes even in very noisy backgrounds. These techniques fail, however, when the target object displays varying size, shape, and brightness. Pattern-matching or template-matching methods generally use template/pattern image(s) that are compared with test images pixel by pixel. The image regions where the distance between the template and the pixels in the image are lower than a predetermined similarity threshold level are labeled as a target (Eq. 5).

(4) $$\begin{array}{l} D_{\left(x,y\right)}=\frac{1}{255\cdot w\cdot h}\sum_{i=1}^{w}\sum_{j=1}^{h}{|I}_{\left(x+i,y+j\right)}-T_{\left(i,j\right)}| \end{array}$$

(5) $$\begin{array}{l} \mathrm{Sim}=\text{1}-D_{\text{(}x\text{,}y\text{)}} \end{array}$$

Here, *D*_(_*_x,y_*_)_ gives the Euclid distance of pixel at *P*_(x,y)_ in the image; *w* and *h* show width and height of the template image in pixel number; the number 255 indicates the bit depth of the pixel for 8-bit images (2^8^ − 1 = 255); *I*_(_*_x_*_+_*_i,y_*_+_*_j_*_)_ indicates brightness level of the pixel at *P*_(_*_x_*_+_*_i,y_*_+_*_j_*_)_ in the test image; and *T*_(_*_i,j_*_)_ gives brightness level of the pixel at *P*_(_*_i,j_*_)_ in the template. An object is counted when the similarity (*Sim* in Eq. 5) is higher than a predetermined threshold. Lowering the similarity threshold increases the detectable particle range; however, it also increases false positive detection (Figure S5A in Supplementary Material). One solution to the problem of target variability is to use multiple template matching (MTM). MTM uses multiple templates designed to as many as possible particles with different sizes and shapes (Figures S5B,C in Supplementary Material) to increase matching rate.

We examined a 200% scale SEM image (Figure S6 in Supplementary Material) to test the accuracy of MTM. We designed four templates with different sized spherical dots (5 × 5, 7 × 7, 9 × 9, and 11 × 11 shown in Figure S6A in Supplementary Material). We predetermined similarity thresholds (80% for 5 × 5, 77% for 7 × 7, 74% for 9 × 9, and 70% for 11 × 11) and background thresholds (brightness levels at 55, 60, 65, and 70) for the patterns. As shown in Figure S6B in Supplementary Material, the detectable numbers of calcified particles were increased by the combined use of all four templates; however, the total accumulated number of identified particles remained less than that of FpPL (MTM, 77.2% for matched particles, 18.8% for false negatives, 4% for false positives; FpPL, 89.1% for matched particles, 7.3% for false negatives, and 3.6% for false positives). Furthermore, the computational time required to complete the process for this method took approximately 5 times longer (10.28 ± 06 s, obtained by analyzing same image 5 times) than the time needed for FpPL (1.98 ± 05 s).

### FpPL Analyses of Aortic Valve Leaflets Reveals Asymmetry of Particle Distribution that Corresponds to CAVD Progression

**Figure 6:**
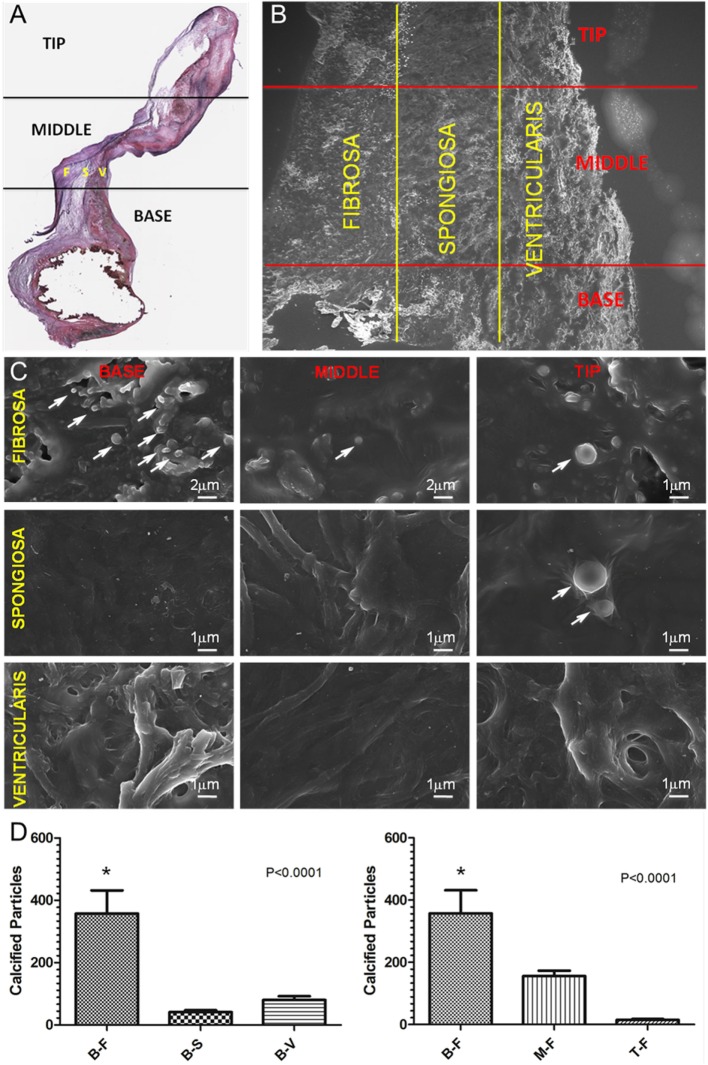
**FpPL quantification of particle distribution in CAVD leaflets**. **(A)** van Gieson stained histological section of a CAVD leaflet indicating tip, middle, and base regions and fibrosa (F), spongios (S), and ventricularis (V) layers. **(B)** Low magnification SEM image of a CAVD leaflet showing nine analyzed regions. **(C)** High magnification SEM images of nine CAVD leaflet regions showing calcified particles (arrows). **(D)** Quantification of calcified particles from CAVD leaflet regions – base fibrosa (B-F), base spongiosa (B-S), base ventricularis (B-V), middle fibrosa (M-F), and tip fibrosa (T-F). Error bars represent SEM (*N* = 3 donors, *n* = 9–11 images per region per donor).

After development and validation of the FpPL approach using synthetic and carotid artery images, we applied the quantification method to CAVD leaflets obtained from patients undergoing aortic valve replacement surgery. Histological sections were divided into base, middle, and tip regions, each corresponding to approximately one-third of the total leaflet length and van Gieson staining identified the three leaflet layers: the fibrosa (F), the spongiosa (S), and the ventricularis (V) (Figure 6A). The resultant nine leaflet portions were then imaged by SEM (Figure 6B) with high magnification scans revealing the presence of calcified particles (Figure 6C, arrows). Quantification of the particles from 9 to 11 images from each of the 9 regions (Figure 6D) revealed significantly more particles located within the base fibrosa (B-F) region compared with the base spongiosa (B-S), and base ventricularis (B-V) (*p* < 0.0001), and within the fibrosa region across the leaflet, the most particles were identified in the base (B-F) compared with the middle (M-F) and tip (T-F) (*p* < 0.0001).

## Discussion

Calcific aortic valve disease initiates at the base of aortic valve leaflets in the fibrosa layer adjacent to the aortic outflow tract. Reversibility of advanced calcification remains unattainable; therefore, detection and treatment strategies must address the early stages of disease before gross remodeling severely hampers valve function (25). Recent analyses showed the presence of small calcified particles in cardiovascular tissues, even in regions without large calcifications, suggesting that these particles may be involved in disease initiation (19). We hypothesized that if these particles do represent early CAVD, they would exhibit regional asymmetry that corresponds to the disease asymmetric development. In order to quantify the distribution of the particles, we had to develop a method to recognize them within relatively low contrast, cluttered SEM images.

Our method, the FpPL, could more accurately detect particulate objects located in an image with background artifacts/textures than standard ImageJ techniques that we examined. FpPL examines the relative difference (subtraction or ratio) between pixel brightness of center part and peripheral zone. In this way, a local threshold is applied to each particulate object. Multiple template matching (MTM) using an array of templates would perform similarly to identify targets of high variability. We prepared four templates to compare with test images (Figure S6 in Supplementary Material). However, the particle detecting efficiency of MTM showed unsatisfactory results. For more precise detection, MTM required many more templates designed for particles varying in size, shape, and brightness, requiring an enormous time to complete the analysis. For these two challenges (to increase detection rate and reduce process time), we prepared multiple resolution images from the original image rather than using multiple templates (Figure 2B). This combinational use of flexible pattern and multiple resolution images detected particles with wide variation in size, shape, and brightness. The accuracy in detecting particles derives from the unique pattern design of FpPL. This design can be adapted for quantification of other types of images and the decreased computational time required for FpPL compared with MTM offers the possibility of image analysis automation. Through the combinational use with the HSV color space, FpPL could target colored particles in the color image stained with multiple dyes. Therefore, this method could be utilized widely to count small particulate objects in noisy backgrounds. Pathological assessments of histological sections often rely on identification of small, colorimetric-stained features within cluttered images. By adapting FpPL to work in HSV color space, automated algorithms could help pathologists identify and quantify features of interest.

By applying the FpPL method to SEM images of aortic valve leaflets, we found that calcified particles predominated in the fibrosa layer at the base of the leaflets, supporting our hypothesis of disease initiation in this specific region. This finding could have profound implications on future research into CAVD detection and treatment. Previous gross histopathological observations noted the regional specificity in calcification of aortic valve leaflets, and the current data indicate that regional changes begin at the earliest stages. FpPL provides a method to quantify these early changes in SEM images – the only method available to visualize the smallest of the calcified particles. The origin and function of these calcified particles remains unknown, but with a method to quantify the distribution of these particles, their association with and changes in response to pathological stimuli and/or therapeutic treatment may provide new insight into the early stages of CAVD, particularly detecting the initiation site of this disease. Though the analyses presented in this study derive from explanted tissues, mechanistic insight may be obtained using FpPL to analyze calcified particles in tissue mimics or *ex vivo* conditions that recapitulate the *in vivo* situation (11). Future studies may build on these analyses to determine the exact role that these particles play in CAVD and examine methods for clinical detection of early particle accumulation in cardiovascular tissues for intervention prior to gross pathologic remodeling (26).

## Author Contributions

KY and PV conceived and designed the experiments. SB, PV, and JH performed the experiments. JH, SCB and PV provided tissue samples. KY analyzed the data. KY, JH, and PV wrote the paper. KY created FpPL algorithm. KY developed software tools. MA edited and revised the manuscript. EA designed and supervised this study.

## Conflict of Interest Statement

KY is an employee of Kowa Company, Ltd. The remaining authors declare no conflict of interest.
