# Supplementary material for: Quantification of Calcified Particles in Human Valve Tissue Reveals Asymmetry of Calcific Aortic Valve Disease Development
Source: Front Cardiovasc Med. 2016 Nov 4;3:44. doi: 10.3389/fcvm.2016.00044 (PMC5095138; doi:10.3389/fcvm.2016.00044)
Supplement: Supplementary file 1 [file presentation_1.pdf]

Supplemental Information

Supplemental experiment 1

[Example 1]

Test image (dot width in pixel number: 1 - 15)

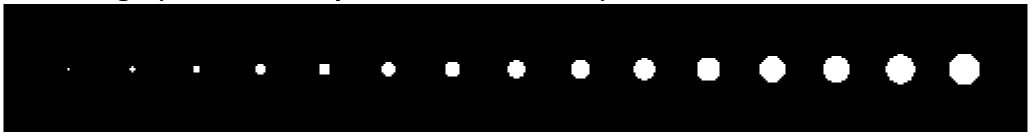

Result

Gap width	Dot width in pixel number														
	1	2	3	4	5	6	7	8	9	10	11	12	13	14	15
0	-	+	+	+	+	-	-	-	-	-	-	-	-	-	-
1	-	+	+	+	+	+	+	-	-	-	-	-	-	-	-
2 (default)	-	+	+	+	+	+	+	+	+	-	-	-	-	-	-
3	-	+	+	+	+	+	+	+	+	+	-	-	-	-	-
4	-	+	+	+	+	+	+	+	+	+	+	+	+	-	-
5	-	+	+	+	+	+	+	+	+	+	+	+	+	+	+
6	-	+	+	+	+	+	+	+	+	+	+	+	+	+	+

+, particle was detected; -, particle was not detected

[Example 2]

Test image (dot width is varied)

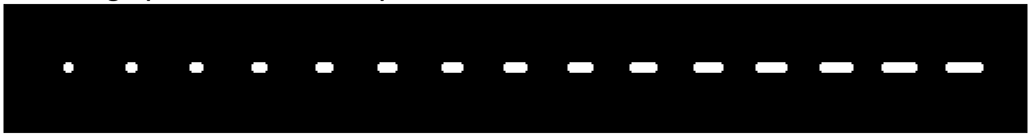

Result

Gap width	Increased pixel number of dot size (width) from original dot size														
	1	2	3	4	5	6	7	8	9	10	11	12	13	14	15
0	+	-	-	-	-	-	-	-	-	-	-	-	-	-	-
1	+	+	+	-	-	-	-	-	-	-	-	-	-	-	-
2 (default)	+	+	+	+	+	+	+	-	-	-	-	-	-	-	-
3	+	+	+	+	+	+	+	+	+	-	-	-	-	-	-
4	+	+	+	+	+	+	+	+	+	+	+	-	-	-	-
5	+	+	+	+	+	+	+	+	+	+	+	+	+	-	-
6	+	+	+	+	+	+	+	+	+	+	+	+	+	+	+

+, particle was detected; -, particle was not detected

[Example 3]

Test image (dots with different sizes: width=2px, 160; 4px, 80; 6px, 40; 8px, 20)

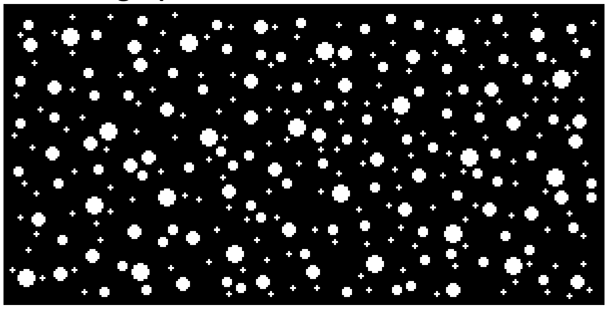

Result

Gap width	Detecting rate of each size of dot (%)			
	Width=2	Width=4	Width=6	Width=8
0	100	100	0	0
1	100	100	100	0
2 (default)	83.8	100	100	100
3	70.6	100	100	100
4	55.6	100	100	100
5	46.3	97.5	100	100
6	39.4	95	100	100

Light blue, no particle was detected
Orange, all particles were detected
Green, particles were partially detected

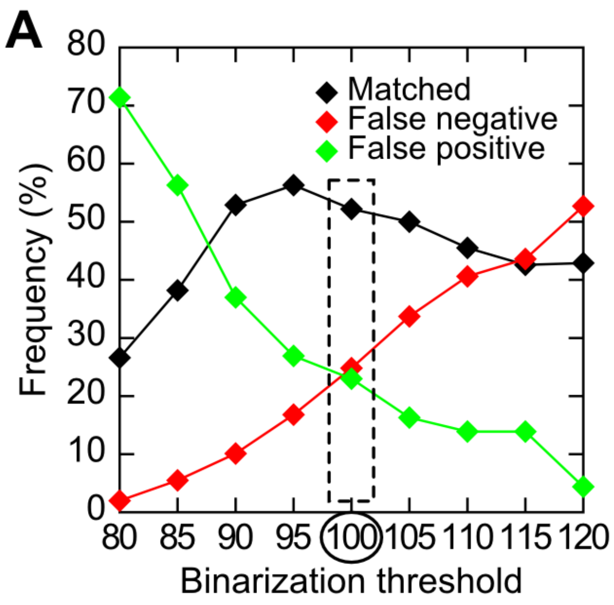

Figure S1. Determining appropriate parameters for the basic particle labeling (BPL) method. **A**, BPL required a binarizing threshold on the brightness level. The value should be determined by maximizing the detection rate (black diamond), while minimizing false negatives (red diamond) and false positives (green diamond). The appropriate threshold value was found to be 100 in our case (box). **B**, In some case noise would appeared as dots when binarization threshold was applied and could be counted as target particles. We overcame this noise by applying a threshold for the minimum pixel size required for an object to be labeled as a particle. For our case we used 3 pixels as the lower limit to determine particles.

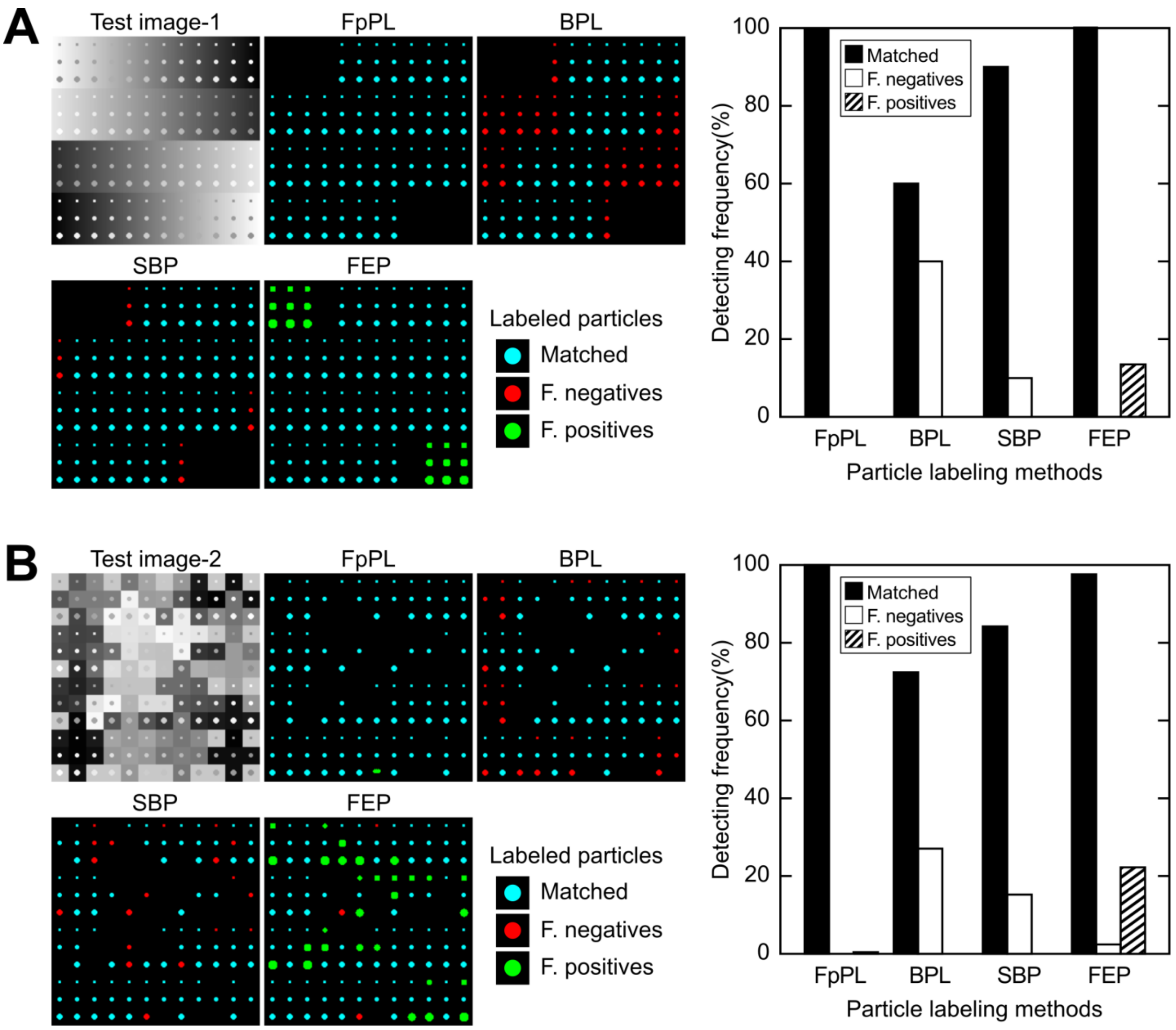

Figure S2. Flexible pattern particle labeling (FpPL) showed highest particle detection rate using synthesized bright dots in noisy background.

A, test image-1 (top-left) and **B**, test image-2 (top-left). Labeled dots by each labeling method were colored cyan for matched particles, red for false negatives (seen only in reference) and green for false positives (seen only in labeled results). Bar graphs show frequency of matched particles (black bars), false negatives (white bars) and false positives (hatched bars).

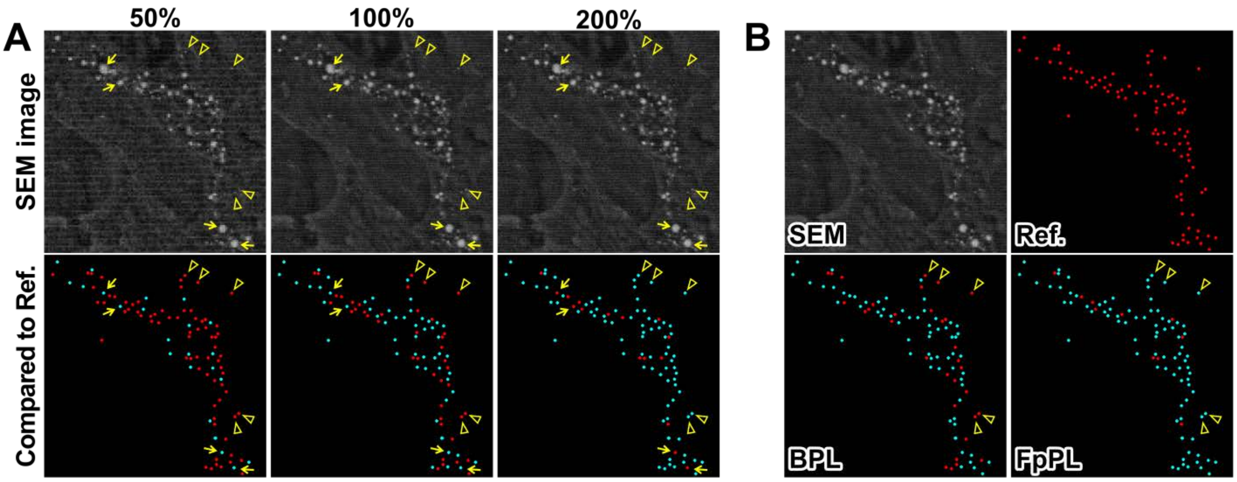

Figure S3. Analyzing enlarged image greatly increased detection of very small and dark calcified particles by FpPL.

A, multiple scaling images were prepared (top-left, 50%; top-center, 100% and top-right, 200%). Labeled particles were compared with reference particle image (top-left, Figure S3B) to validate the detection (matched, cyan; false negatives, red, bottom, Figure S3A). Arrowheads indicate very small and dark particles, which were only detected in an enlarged image (200%) by FpPL. Arrows indicate larger calcified particles, which were not recognized in the enlarged image. **B**, Test image (top-left), reference particle image determined by eye (top-right), labeled image by BPL (bottom-left) and labeled image by FpPL (bottom-right) are shown. Arrowheads show very small particles, which were not recognized by BPL.

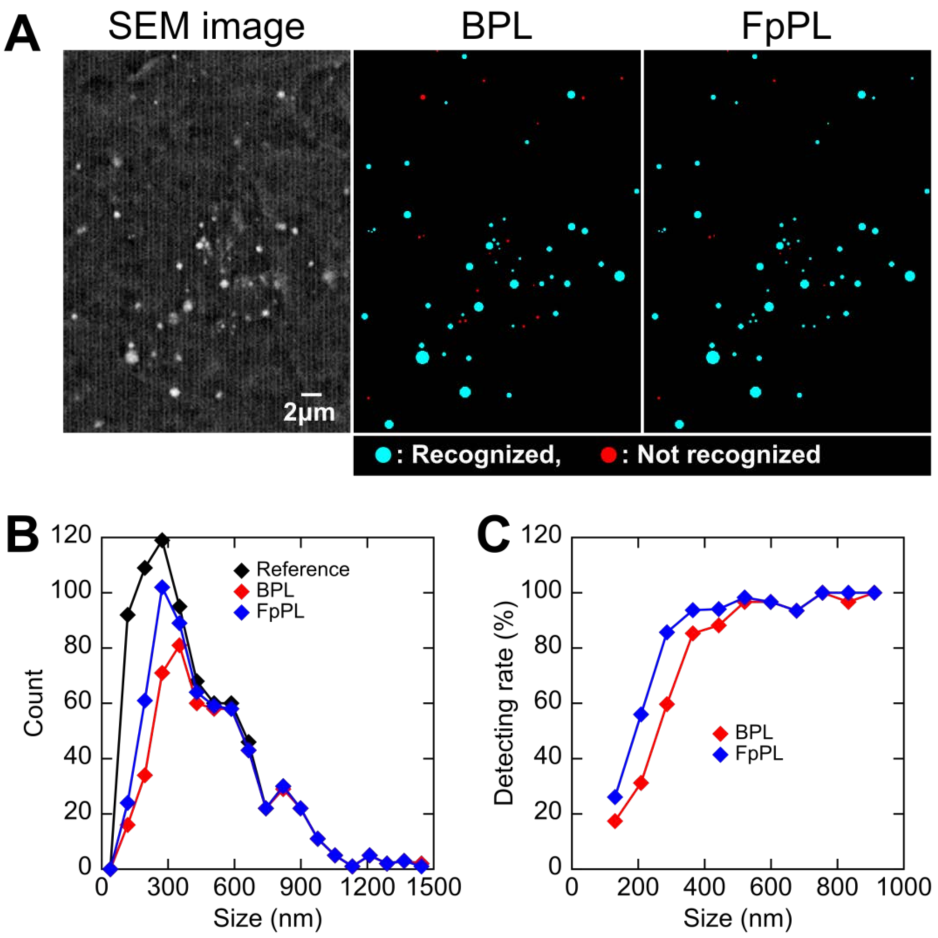

Figure S4. FpPL had higher particle detection rate of small calcified particles than the BPL method.

A, Calcified particles in the SEM images were sized and marked by small red dots, which was used as a reference. The particles labeled by FpPL or BPL were colored light green, and the results were compared with the reference. Matched particles (cyan dots) and false negatives (red dots) are indicated (false positives are not shown). **B**, From a total 756 calcified particles in 5 pictures including 2 different donors, the particle size showed one peak around 300 nm, and particles ranged from 100 nm to 1.5 μm. Size distribution and average size of calcified particles did not exhibit donor variation (donor-1, particle number = 377, average size = 476.4 $\pm$ 267.7 nm; donor-2, particle number = 379, average size = 455.1 $\pm$ 265.5 nm; merged, 465.8 $\pm$ 266.7 nm, particle number = 756). **C**, In both methods detection rates of particles smaller than the peak value (< 300 nm) were decreased; however, FpPL indicated higher detection rate of smaller particles than BPL. The points where detection curves crossed 50% detection rate were 189.1 nm for FpPL and 255.1 nm for BPL.

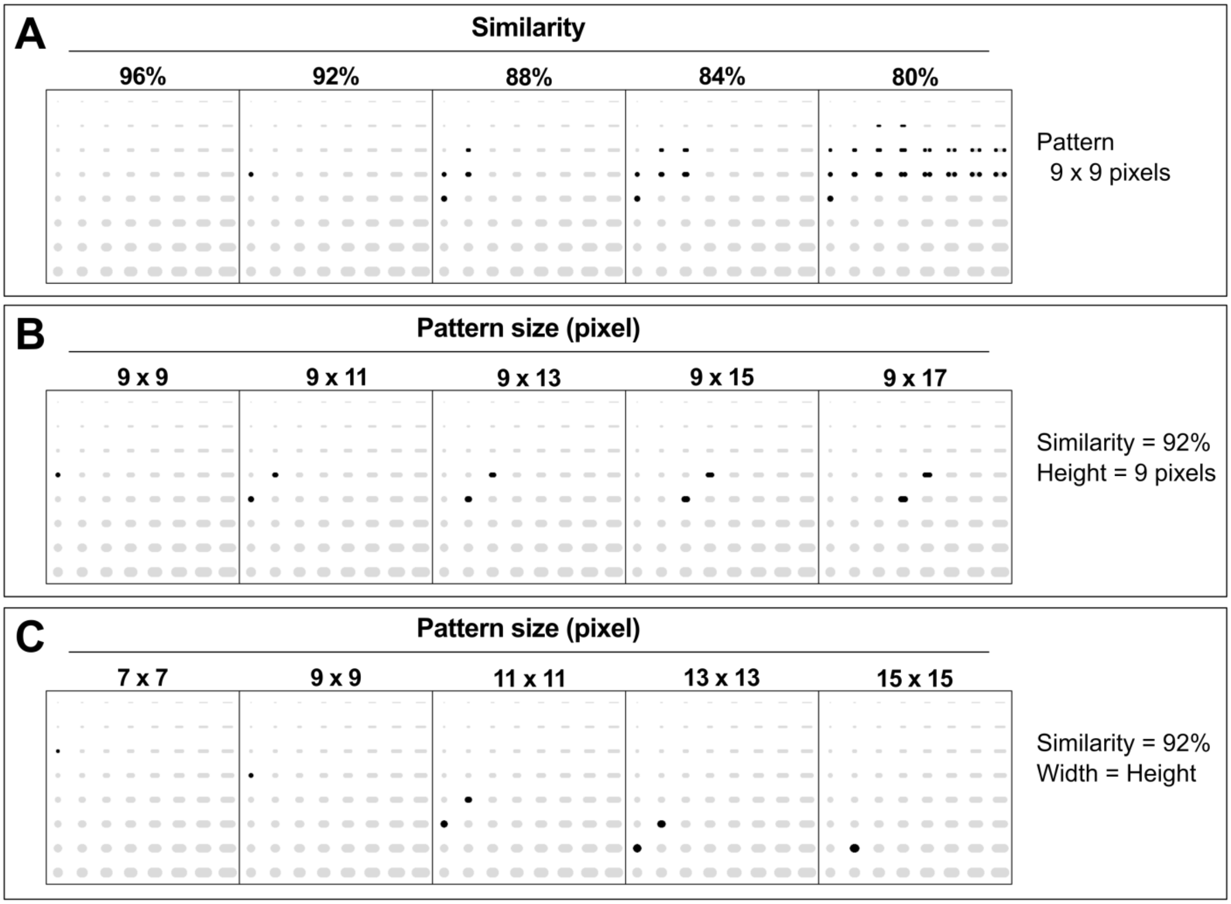

Figure S5. General template matching method showed strict detection of target that restrict countable range in sizes and shapes.

A, In the pattern matching method the similarity threshold between the test pattern and the target particle was key to label targets precisely. The test pattern moves across the image and labels a particle when the similarity between the particle and test pattern is above the similarity threshold. Patterns with 9 x 9 varying sized dots were used for matching. No particle was labeled when the similarity threshold between the test pattern and particles in the synthetic image was 94% (left). The number of labeled particles was increased according to a decrease in similarity threshold. **B**, Test patterns that had 9 pixel heights but with different pixel widths were used to label the synthetic image. Depending on the increase of the width, the detectable range of the particles shifted horizontally in the image. **C**, Patterns with same portion in height and width were used. According to the test pattern size, the detectable range of particles was shifted vertically (moved to downward).

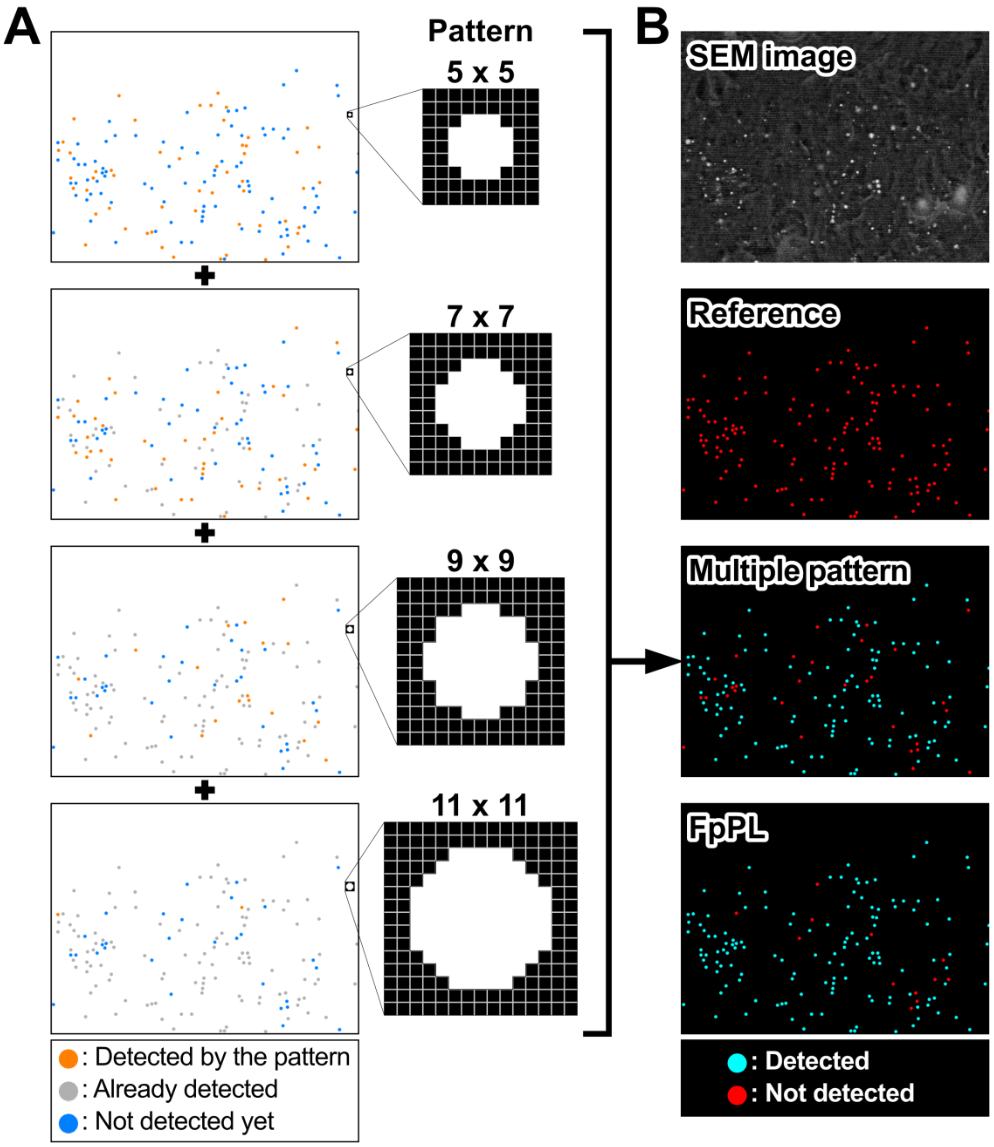

Figure S6. Multiple template matching increased particle detection rate but less accurate than FpPL.

A, To demonstrate how the multiple template pattern matching works, four particle templates were used to detect particles. Each template scanned through the image sequentially in the order shown. The particles detected by the current pattern were colored orange and the particles that were not yet detected were colored blue. Particles already detected by the previous patterns were labeled gray. **B**, Detection accuracies of the multiple pattern matching method and FpPL were also compared. FpPL outperformed the multiple template matching technique, indicating that an insufficient number of template were used. Additional templates require additional computational power..
